# Phagosomal TLR signaling upon *Borrelia burgdorferi* infection

**DOI:** 10.3389/fcimb.2014.00055

**Published:** 2014-05-20

**Authors:** Jorge L. Cervantes, Kelly L. Hawley, Sarah J. Benjamin, Bennett Weinerman, Stephanie M. Luu, Juan C. Salazar

**Affiliations:** ^1^Department of Pediatrics, University of Connecticut Health CenterFarmington, CT, USA; ^2^Division of Infectious Diseases, Connecticut Children's Medical CenterHartford, CT, USA; ^3^Department of Molecular Biology and Biophysics, University of Connecticut Health CenterFarmington, CT, USA; ^4^Department of Immunology, University of Connecticut Health CenterFarmington, CT, USA

**Keywords:** *Borrelia burgdorferi*, toll-like receptors, innate immunity, phagosomal signaling, Lyme disease

## Abstract

Internalization and degradation of live Bb within phagosomal compartments of monocytes, macrophages and dendritic cells (DCs), allows for the release of lipoproteins, nucleic acids and other microbial products, triggering a broad and robust inflammatory response. Toll-like receptors (TLRs) are key players in the recognition of spirochetal ligands from whole viable organisms (i.e., vita-PAMPs). Herein we will review the role of endosomal TLRs in the response to the Lyme disease spirochete.

## Introduction

*Borrelia burgdorferi* (Bb), the causative agent of Lyme disease (Radolf et al., 2012) is the most prevalent vector-borne disease in North America (Levi et al., 2012). In nature, Bb persists asymptomatically in rodent reservoirs, such as the white-footed mouse *Peromyscus leucopus* (Barthold and Philip, 2010). Infection in humans is incidental, and probably represents a dead-end for the bacterium, as the spirochete is not well adapted to the human host.

Bb contains a complex bacterial genome (Fraser et al., 1997), and is composed of different strains and genospecies with differences in infectivity, host range, and tissue tropism (Schutzer et al., 2011; Yang et al., 2013). Different from gram-negative bacteria, Bb does not contain LPS (Takayama et al., 1987), and instead it has a number and variety of lipoproteins, many of them embedded on the spirochete's outer membrane (Bergstrom and Zückert, 2010). A number of well-known borrelial lipoproteins (e.g., OspA) are preferentially expressed at particular stages of the enzootic cycle (Radolf et al., 2012).

Bb also lacks any toxigenic molecules, thus, the clinical manifestations associated with Lyme disease are thought to result from the host's innate and adaptive immune response to the invading spirochete (Radolf et al., 2012). A great amount of research has been done trying to elucidate the mechanism by which Bb causes inflammation, which in some Lyme Disease patients manifests as severe arthritis, carditis, and/or central nervous system disorders (Radolf and Samuels, 2010). Initial studies focused on the role of *Borrelia*'s abundant lipoproteins, which are capable of binding CD14 and Toll-like receptor (TLR) 2/TLR1 heterodimers on the surface of phagocytic cells to induce the production of inflammatory cytokines (Weiss, 2010; Radolf et al., 2012). However, we and others have shown that internalization and degradation of live Bb by monocytes and macrophages within phagosomal compartments, allows for the release of lipoproteins and other microbial products, including RNA and peptidoglycan, eliciting a broader and more complex inflammatory response that can possibly take place on the cell surface of innate immune cells (Salazar et al., 2009; Cervantes et al., 2011). We have called this process “phagosomal signaling.”

TLRs are transmembrane receptors which can recognize various pathogen-associated molecular patterns (PAMPs), including spirochetal lipoproteins and nucleic acids. The interaction between spirochetal PAMPs and the TLRs triggers a variety of intracellular signaling pathways leading to the production of various cytokines, including type I interferons (Salazar et al., 2009; Cervantes et al., 2011). This review focuses on the role of endosomal TLRs in Bb-mediated phagosomal signaling.

## *B. burgdorferi* infection and the innate immune response

While studies have shown that neutralizing antibodies are important in host defense against Bb infection, the innate immune system is now known to have a critical role in spirochetal recognition and clearance from infected blood and tissues (Barthold et al., 1992). Monocytes, dendritic cells, macrophages, Natural Killer cells (NK-cells), NK-T cells, and polymorphonuclear cells (PMNs), all contribute to generate a coordinated and robust response to Bb infection (Salazar et al., 2003; Moore et al., 2007). We have proposed a model, where this response is initiated through recognition of specific Bb ligands during phagocytosis, primarily by the activation of endosomal TLRs (Moore et al., 2007; Petzke et al., 2009; Salazar et al., 2009; Cervantes et al., 2011). In this section we will briefly describe the role of NKT cells, PMNs, monocytes/macrophages and DCs in spirochetal recognition.

### Natural killer T (NKT) cells

NKT cells play an important role in the regulation of the inflammatory response during Bb infection (Lee et al., 2010). In one study, Bb-infected NKT-depleted-mice had greater inflammation in infected joint tissues, as well as decreased pathogen clearance (Tupin et al., 2008). This finding is not surprising, given that Antigen-presenting cells (APCs) activate NKT cells through presentation of a diacylglycerol glycolipid (Olson et al., 2009). Upon activation with Bb, NKT cells upregulate of IFNγ to activate and differentiate macrophages (Olson et al., 2009). It is not know whether NKT cells have a role in the pathogenesis of Lyme arthritis in humans.

### Polymorphonuclear cells (PMNs)

The neutrophil is an essential element of the inflammatory response to the Lyme disease spirochete in both skin and joints (Salazar et al., 2003; Radolf and Samuels, 2010). Indeed, PMNs have been shown to play a significant role in the development of Lyme arthritis in experimentally infected mice (Nardelli et al., 2008; Codolo et al., 2013). In humans, PMNs are the primary cell type present in joint fluids from LD patients diagnosed with acute arthritis. PMNs are responsible for the production of several inflammatory cytokines, various chemokines, and stimulating factors that are likely to contribute to inflammation in the joints by inducing macrophage migration and differentiation and T-cell activation (Georgilis et al., 1991; Brown et al., 2003; Mantovani et al., 2011).

### Monocytes

**Figure 1:**
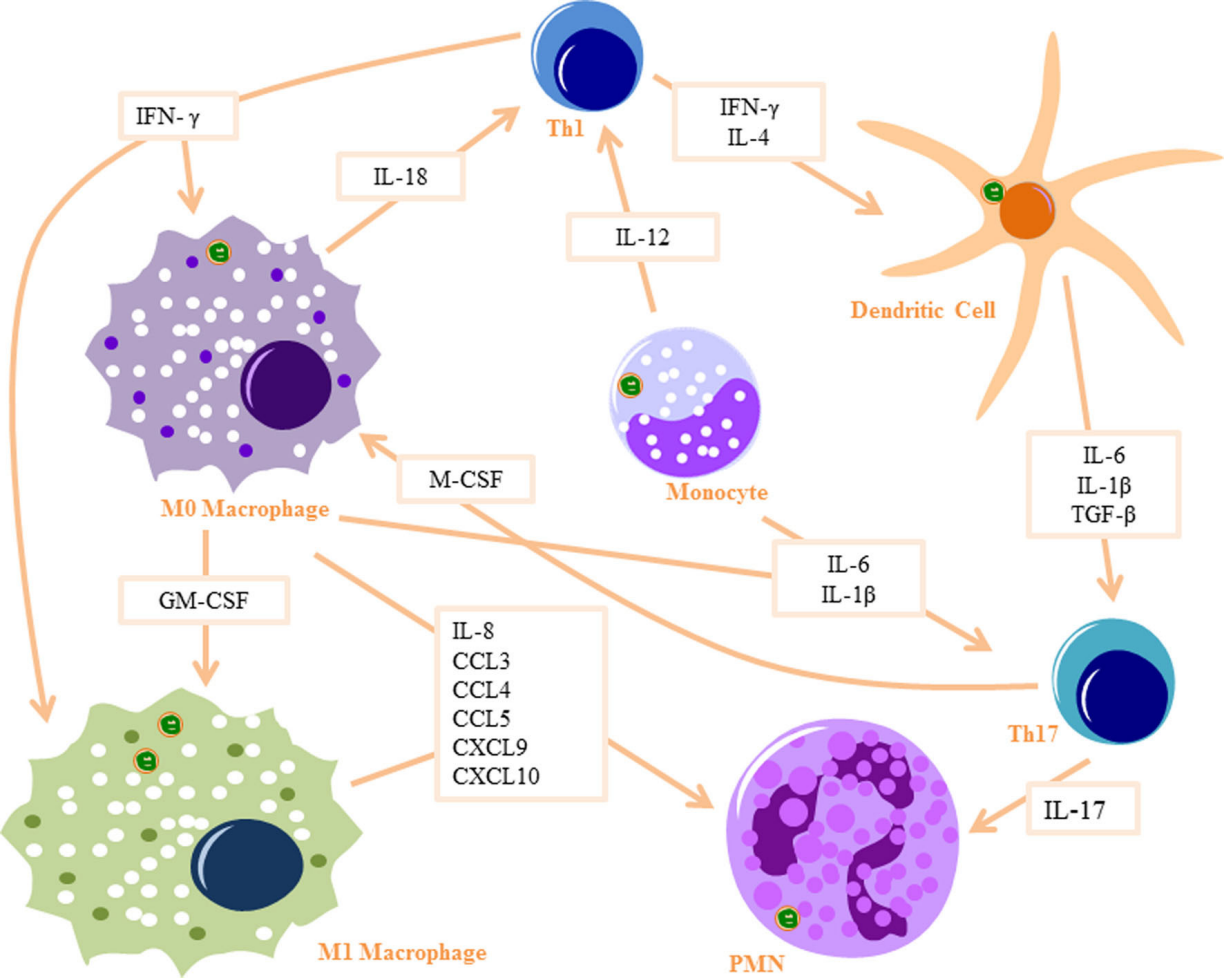
***Innate signaling cascade in response to Bb Phagocytosis*: Monocytes produce IL-12 to activate Th1 cells and IL6 and IL-1β to activate Th17 cells.** Monocytes differentiate into macrophages when stimulated with IFN-γ (produced by Th1 cells) and M-CSF (produced by Th17 cells) and differentiate into dendritic cells when stimulated with IL-4 and IFN-γ. M0 macrophages produce IL-18 to activate Th1 cells and IL-6 and IL-1β to activate Th17 cells, as well as several PMN recruitment chemokines. They also differentiate into M1 macrophages when stimulated with GM-CSF and IFN-γ. Dendritic cells produce IL-6, IL-1β, and TGF-β to activate Th17 cells. Th17 cells produce IL-17, which is a strong PMN attractant. Part of images from Motifolio drawing toolkit (www.motifolio.com) were utilized in the figure preparation.

Monocytes are thought to have an important role in the production of proinflammatory cytokines during Bb infection (Salazar et al., 2009). Phagocytosis of Bb by human monocytes activates signaling cascades, which induce transcription of proinflammatory cytokines, including IL-6, TNF-α, and IL-12 (Salazar et al., 2003; Cruz et al., 2008; Salazar et al., 2009) (Figure 1). IL-6 activates T helper cells and induces Th17 differentiation, which in turn can recruit PMNs through IL-17 production (Burchill et al., 2003). TNF-α has the ability to increase vascular permeability at the infection site, resulting in increased infiltration of PMNs and other innate immune cells. IL-12 production by the monocytes is likely to induce Th1 differentiation and increased secretion of IFNγ (Biswas and Mantovani, 2010). IFNγ plays an important role in M1 macrophage differentiation at infection sites (Mosser and Edwards, 2008). Monocytes also upregulate pro-IL-1β in response to Bb (Cruz et al., 2008), which when cleaved into IL-1β by caspase-1 can also induce Th17 differentiation (Chung et al., 2009). Bb is also known to induce monocyte inflammatory cell death through an intrinsic signaling pathway (Cruz et al., 2008), a mechanism which possibly leads to the recruitment of other immune cells to Bb-infected tissues.

### Macrophages

Bb phagocytosis by macrophages results in increased transcription of IL-1β, IL-6, TNF-α, and type I IFNs (Strle et al., 2009). Bb-infected macrophages also produce a number of PMNs chemoattractants, including CCL3, CCL4, CCL5, CXCL9, and CXCL10 (Gautam et al., 2012). Macrophages also produce IL-18 in response to Bb infection (Dennis et al., 2006; Oosting et al., 2011), which in turn induces IFNγ production by Th1 cells, driving M1 macrophage polarization (Mosser and Edwards, 2008). M1 macrophages upregulate iNOS and reactive oxygen species (Biswas and Mantovani, 2010), which are essential in clearance of Bb (Boylan et al., 2008). Phagocytosis of Bb by macrophages results in significant production of the anti-inflammatory cytokine IL-10, to mediate resolution of the already initiated cytokine response, a phenomenon which may play a critical role in Lyme disease severity and arthritis development (Lazarus et al., 2008; Gautam et al., 2012; Chung et al., 2013).

### Dendritic cells (DCs)

DCs are enriched in early Lyme disease skin lesions (*erythema migrans*) (Salazar et al., 2003) and are amongst the first immune cells to come into contact with Bb in the skin (Mason et al., 2014). Phagocytosis of Bb activates DCs by inducing expression of CD83 and upregulating expression of CD40, CD80, CD86, and HLA-DR (Suhonen et al., 2003) and lead to increased transcription of proinflammatory cytokines, including IL-6, IL-1β, and TNF-α (Petzke et al., 2009). TGF-β is also produced by DCs after Bb infection, a cytokine which in turn induces Th17 differentiation (Chung et al., 2009). The PMN chemotactic factor IL-8 (Dennis et al., 2006), as well as a number of other PMN chemoattractants (Hartiala et al., 2007) are produced by DCs following Bb infection. Upon contact with Bb in the skin, DCs migrate to the lymph nodes where they present antigens to T cells and induce adaptive immune responses (Mason et al., 2014). Similarly to Bb-stimulated macrophages, IL-10 is also elicited from Bb-infected DCs (Chung et al., 2013). IL-10 down-regulates macrophage activation, decreases the production of proinflammatory mediators, and suppresses phagocytosis-associated events that are important for mediating both innate and adaptive immune responses by APCs (Chung et al., 2013).

## Murine vs. human

The development of a murine model to study Lyme disease has greatly advanced our understanding of the cellular and humoral responses to Bb. All inbred stains of laboratory mice are susceptible to Bb infection, although each strain differs in their disease severity. C3H/He and Balb/c mice are more susceptible while C57BL/6 and SJL mice tend to be more resistant to Bb infection (Barthold and Philip, 2010). Differences in the arthritis severity of these two strains might not involve discrepancies in bacterial clearance mechanisms, as both harbor similar numbers of spirochetes within their ankle joints (Ma et al., 1998). C3H cells have been reported to produce higher levels of NFκ-B cytokines than C57BL/6 upon stimulation with purified borrelial lipoproteins (Ganapamo et al., 2003). However, differences in acquired immune responsiveness after whole Bb infection are still not well understood. Furthermore, studies on the role of NFκ B-dependent cytokines in arthritis development have yielded conflicting results (Wooten and Weis, 2001). The susceptible strains produce higher IgG titers and tend to have more severe arthritis and carditis with similar manifestations to human infection (Barthold and Philip, 2010; Weiss, 2010). Importantly, no mouse strain develops the hallmark skin lesion *erythema migrans* caused by the inflammatory response elicited by the spirochetes, nor neurological disease, making the murine Lyme model an imperfect model for human Lyme disease. Rhesus monkeys infected with Bb develop neuroborreliosis and *erythema migrans* in addition to arthritis, thus making the disease most similar to human infection. Costs and difficulties in genetic manipulation make the murine model more commonly used over the primate model (Barthold and Philip, 2010; Radolf et al., 2012).

Lyme carditis is caused primarily by the infiltration of monocytes and macrophages in Bb infected mice (Weiss, 2010). The genetic background of the experimentally infected murine model influences the susceptibility to the development of carditis, although the root of the diversity remains unknown. IFNγ has been shown to locally modulate Lyme carditis and enhance the phagocytic capacity of macrophages (Olson et al., 2009). Macrophages are a critical part of the response to Bb, especially for the control of spirochetal numbers in the heart (Behera et al., 2006; Olson et al., 2009). In humans, symptomatic Lyme carditis is rare and usually resolves with antibiotic therapies (Krause and Bockenstedt, 2013), although recently it has been shown to be associated with mortality rates higher than previously reported (CDC, 2013).

The field of Lyme arthritis has gained much insight from the use of C3H mouse models due to the robust phenotype that mimics much of human Lyme disease arthritis complications (Barthold and Philip, 2010). However the spectrum of pathology observed in humans is not observed in the murine model (Steere et al., 1987). It has also been reported that there is a lack of T cell involvement in mice, which is contradictory to the observations of some human patient reports, suggesting the importance of Th1 and γ/δ T cells (Duray, 1989; Vincent et al., 1996; Gross et al., 1998; Roessner et al., 2003). A select group of Lyme arthritis patients experience persistent arthritis even following an appropriate course of antibiotic therapy (Shin et al., 2007). Investigation of this cohort of patients' synovial fluid suggests there is a possible dysregulated inflammatory response that presents with increased IL-1β, IL-6, and IFNγ (Shin et al., 2007) and chemokines CXCL9 and CXCL10 (Shin et al., 2007). IFN-responsive genes have been reported to be strongly up-regulated within the joints of Bb-infected C3H mice, but not in mildly arthritic C57BL/6 mice (Crandall et al., 2006). Nevertheless, bone marrow-derived macrophages from both C3H and C57BL/6 mice induce IFN-responsive genes following Bb stimulation (Miller et al., 2008a), and this expression appears to require a functional type I IFN receptor (Miller et al., 2008b). The arthritis observed in C57BL/6 mice is modulated by the production of IL-10 from CD4^+^ T cells and macrophages (Lazarus et al., 2008). IL-10 regulates the expression of IFNγ and production of CXCL9 and CXCL10 (Lazarus et al., 2008; Sonderegger et al., 2012), and produce a similar phenotype of persistent arthritis patients (Shin et al., 2007).

TLR expression varies slightly between humans and mice, with ten human TLRs, and twelve murine functional TLRs identified to date (Gosu et al., 2012). TLR1-9 are conserved between the two species (Kawai and Akira, 2010). TLR8 is non-functional in mice because it lacks five amino acids (Liu et al., 2010). However, recently, a murine model expressing functional human TLR8 on a C57BL/6 background has been generated (Guiducci et al., 2013). TLR10 is also non-functional in mice due to a retrovirus insertion (Gosu et al., 2012). TLR11-13 are present in mice, but absent in humans (Kawai and Akira, 2010). In humans, TLR7 is mainly co-expressed with TLR9 on B cells and plasmacytoid DCs and TLR8 is nearly absent in this cell line; whereas TLR8 is highly expressed on monocytes/macrophages and myeloid DCs (Hornung et al., 2002; Cervantes et al., 2012). Differences in the inflammatory responses to Bb observed between human and mice, could be explained by variations in TLR- signaling (Petnicki-Ocwieja et al., 2013). Newly generated humanized mouse models would allow for future studies regarding the role of human TLRs in Lyme disease clinical manifestations and severity of disease.

## Phagocytosis of *B. burgdorferi*

Phagocytosis is an important component of innate immunity to the Lyme disease spirochete. Uptake and degradation of the bacterium results in the induction of intracellular signals leading to the generation of cytokines, antigen processing and presentation, which ultimately leads to the development of acquired immunity (Greenberg and Grinstein, 2002; Moore et al., 2007).

Delivering individual PAMPs in experimental systems fails to mimic the natural processes of innate immune activation by TLRs in response to a live organism. In the case of endosomal TLRs, ligands are usually delivered in a complex with a cationic polymer such as polyethylenimine or DOTAP (Cervantes et al., 2013; Love et al., 2014). The natural agonists for endosomal TLRs are, however, an integral part of live pathogens and as such, are not directly accessible to receptors but after the whole organism has been degraded in the endolysosomal compartment (Vance et al., 2009). The immune system responds more robustly to viable microorganisms than it does to dead organisms. Blander and colleagues found that viable bacteria, but not killed ones, contain a class of PAMPs which they coined as vita-PAMPs (Sander et al., 2011). These vita-PAMPs, such as mRNA, signify microbe viability to the innate immune system (Sander et al., 2011). Pathogen and host cell-derived material associated with pathogen nucleic acids have to be taken up into the endolysosomal compartment, where degradation allows the nucleic acids to become available for TLR binding. The detection of vita-PAMPs and conventional PAMPs could interact with multiple PRRs within the phagosome and/or the cytosol and have a crucial role in antimicrobial immunity (Sander et al., 2011).

**Figure 2:**
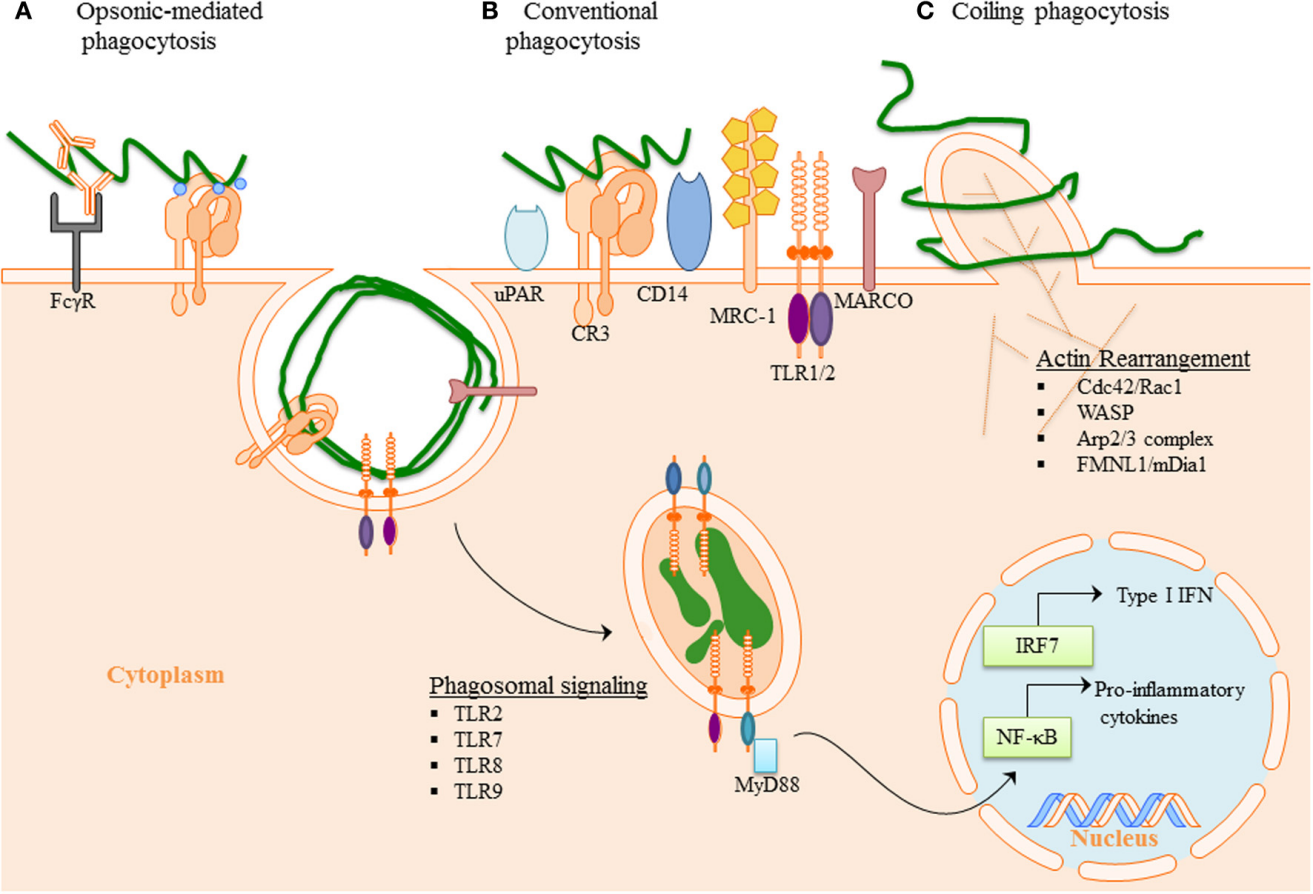
**Phagocytosis of *B. burgdorferi*: (A) Opsonic-mediated phagocytosis—complement factors, such as C3b and iC3b, bound to the surface of Bb can interact with complement receptors and mediate phagocytosis.** Additionally, the immune cells Fc-receptors have the ability to bind to the opsonic antibodies that coat Bb and internalize the pathogen. **(B)** Conventional phagocytosis—the direct interaction of surface receptors with Bb, such as integrins and C-type lectins, allows for tether of the spirochete to the cell surface. Various PRRs induced signal cascade initiates formation of the phagocytic cup and spirochete engulfment. **(C)** Coiling phagocytosis—the preferred mechanism of spirochete internalization in which the phagocytic cell uses filopodial protrusions that capture Bb. The filopodial enwrap the spirochete and convert into coiling pseudopods. During this dynamic process FMNL1, mDai1 Arp2/3 complex, and WASP are involved in actin rearrangement of the cell, which then facilitates subsequent phagocytosis of Bb. Following internalization of Bb, the spirochete is degraded within the phagosome thus exposing additional PAMPs to PRR with in the phagosome. The phagosomal signals initiated by Bb generates a robust inflammatory response, including the induction of pro-inflammatory genes and Type I IFNs.

Professional immune phagocytes, such as monocytes, macrophages, DCs (Benach et al., 1984a; Filgueira et al., 1996; Moore et al., 2007) as well as other various cell types (ex. murine microglia, chondrocytes, synovial, and L929 fibroblast) have been demonstrated to internalize Bb (Franz et al., 2001; Kuhlow et al., 2005; Behera et al., 2008; Chmielewski and Tylewska-Wierzbanowska, 2010). The initiation of phagocytosis requires interaction of phagocytic receptors (other than CD14 and TLRs) located on the surface of innate immune cells with surface molecules of Bb innate immune cells (Shin et al., 2008; Sahay et al., 2009), and internalization of Bb by murine, rat and rabbit macrophages can occur in the presence (Fc-mediated phagocytosis) (Figure 2A) or absence of opsonic antibodies (Figure 2B) (Benach et al., 1984b; Montgomery et al., 1994). Bb is able to activate both the classical and alternative complement pathways (Kochi and Johnson, 1988). C3b can either bind to the surface of bacteria and facilitate internalization of the spirochete by opsonization or C3b or proceed to membrane attack complex formation and lysis of the bacteria by depositing downstream components into the cell wall. The spirochete has evolved mechanisms that enable them to evade complemented-mediated lysis (Fraser et al., 1997), such as expression of complement regulator-acquiring surface proteins (CRASPs). CRASPs act as binding sites for the complement inhibitor factor H and factor H-like protein (Hellwage et al., 2001; Kraiczy et al., 2001, 2004) and cleave bound serum complement protein C3b into the serum opsonin inactivated C3b (iC3b) (Figure 2A).

Complement Receptor 3 (CR3) (integrin α_M_β_2_, CD11b/CD18) was demonstrated to directly bind Bb and the presence of complement enhanced spirochetal binding (Cinco et al., 1997; Garcia et al., 2005). ICAM-1, iC3b and fibrinogen, known CR3 protein ligands, have been shown to interact with the I-domain of CR3 (Humphries, 2000). Only recently, Hawley et al. identified CR3 to be a phagocytic receptor in murine macrophage and human monocyte (Figure 2B). This study revealed that CR3 requires cooperation of the GPI-anchored protein, CD14 for the internalization of unopsonized Bb and that CR3-mediated phagocytosis of Bb occurs in a Myeloid differentiation primary response gene 88 (MyD88)-independent manner, thus suggesting the involvement of additional receptors in MyD88-dependent Bb phagocytosis (Hawley et al., 2012). CD14 is involved in translocation of CR3 to the lipid rich microenvironments known as lipid rafts following interaction with live Bb (Hawley et al., 2013). The findings suggest that CD14 interacts with the C-lectin domain of the integrin to induce crosslinking of the integrin and efficient internalization of Bb. Additionally, MyD88-independent inflammatory pathways have been reported following interaction of the spirochete with the integrin α_3_β_1_ on primary human chrondrocytes and may be a mechanism directly relevant to the development of arthritis (Behera et al., 2008). Moreover, urokinase receptor (uPAR, CD87) has been shown to facilitate clearance of Bb (Figure 2B), and provides an area for additional investigation as to the mechanistic involvement in bacterial clearance (Hovius et al., 2009).

Scavenger receptors comprise a group of unrelated transmembrane surface molecules with relatively promiscuous ligand binding such as scavenger receptor A (SR-A), MARCO (Macrophage receptor with Collagenouse structure) and CD36 (Areschoug and Gordon, 2009). This promiscuity allows scavenger receptors to mediate uptake of a wide range of pathogens including bacteria (Peiser et al., 2000; Thelen et al., 2010), yeast, viruses and parasites (Mukhopadhyay and Gordon, 2004), as well as removal of dead cell material by both macrophages and DCs (Brencicova and Diebold, 2013). Little is known about their natural ligands and the structural basis for ligand binding of bacteria. CD36 and MARCO can interact with TLR2 and CD14, regulating NFκ-B cytokine responses (Bowdish et al., 2009; Jordo et al., 2011). MARCO expression appears to be MyD88 dependent in mice, and MARCO mediated phagocytosis of Bb seems an important mechanism for TRIF signaling (Petnicki-Ocwieja et al., 2013). The mannose receptor has been demonstrated to bind to Bb (Cinco et al., 2001), but its role in Bb phagocytosis has not yet been investigated. Unlike the mannose receptor or CR3 that reconstitute phagocytosis in non-phagocytic cells, expression of SRA or MARCO confers only binding, without significant internalization of Gram positive or Gram negative bacteria (Underhill and Ozinsky, 2002). In macrophages, the scavenger receptors MARCO and SR-A are involved in uptake of CpG ODN and influence TLR9-mediated IL-12 induction with MARCO enhancing its production (Jozefowski et al., 2006).

Coiling phagocytosis is the preferred mechanism of Bb uptake, accounting for approximately 60 to 70% of phagocytosis (Rittig et al., 1992, 1998; Naj et al., 2013). Coiling phagocytosis was first described as the phagocytic mechanism used to internalized *Legionella pneumophila* (Horwitz, 1984) where a unilateral pseudopod bends around the bacteria in a hook like fashion. Phagocytosis is a complex mechanism where F-actin polymerization occurs to reorganize the membrane to internalize Bb (Chimini and Chavrier, 2000; Cruz et al., 2008). The Rho GTPases, Cdc42 and Rac1 regulate the actin dynamics (Chimini and Chavrier, 2000; Linder et al., 2001). WASP (Wiskott-Aldrich syndrome protein) and the Arp2/3 complex are mostly involved in branched actin network, to reorganize to the pseudopod intertwining Bb (Amann and Pollard, 2001; Higgs and Pollard, 2001; Linder et al., 2001) (Figure 2C). The formins, FMNL1 and mDia1, are actin nucleating proteins that influence formation of unbranched actin filaments and regulate coiling phagocytosis of Bb by primary human macrophages (Naj et al., 2013). In neutrophils, mDia1 is required for both CR3-mediated and FcγR- mediated phagocytosis (Shi et al., 2009).

Much focus has been directed to the downstream signals initiated by TLRs following internalization of Bb, especially those involving the adaptor molecule MyD88. Mice deficient in MyD88 have increased bacterial burdens but normal antibody production in comparison to wild-type (WT) mice infected with Bb (Liu et al., 2004). MyD88 deficient macrophages have a significant defect in their phagocytic ability of Bb, roughly 50% reduction compared to the WT macrophages, supporting observations with phagocytosis and killing of bacteria, rather than a previously suggested defect in degradation in phagolysosome (Blander and Medzhitov, 2004; Yates and Russell, 2005; Shin et al., 2009).

## Phagosomal recognition of *B. burgdorferi* ligands

Although there is plenty of evidence showing that formerly “outer membrane-associated TLRs” such as TLR2 and TLR4 are also recruited to the endosome (McGettrick and O'Neill, 2010; Gangloff, 2012; Brandt et al., 2013), the classic “endosomal nucleic acid-sensing TLRs” comprises TLR3, TLR7, TLR8, and TLR9 (Brencicova and Diebold, 2013). TLR 7, TLR8, and TLR9 depend on the endoplasmic reticulum (ER)-resident protein UNC93B1 for trafficking from the ER via the Golgi to the endolysosomal compartment (Brinkmann et al., 2007; Kim et al., 2008; Lee et al., 2013). UNC93B1 physically associates with the endosomal TLR (McGettrick and O'Neill, 2010; Itoh et al., 2011) and it appears to be essential for TLR8-mediated signaling (Itoh et al., 2011). Other factors involved in endosomal TLR trafficking are the ER chaperone GP96 and PRAT4A (McGettrick and O'Neill, 2010; Lee et al., 2013). Furthermore, trafficking of endosomal TLRs is also affected by recruitment of adaptor protein complexes, which are not only TLR-specific (Lee et al., 2013) but cell specific as well (Sasai et al., 2010; Henault et al., 2012).

**Figure 3:**
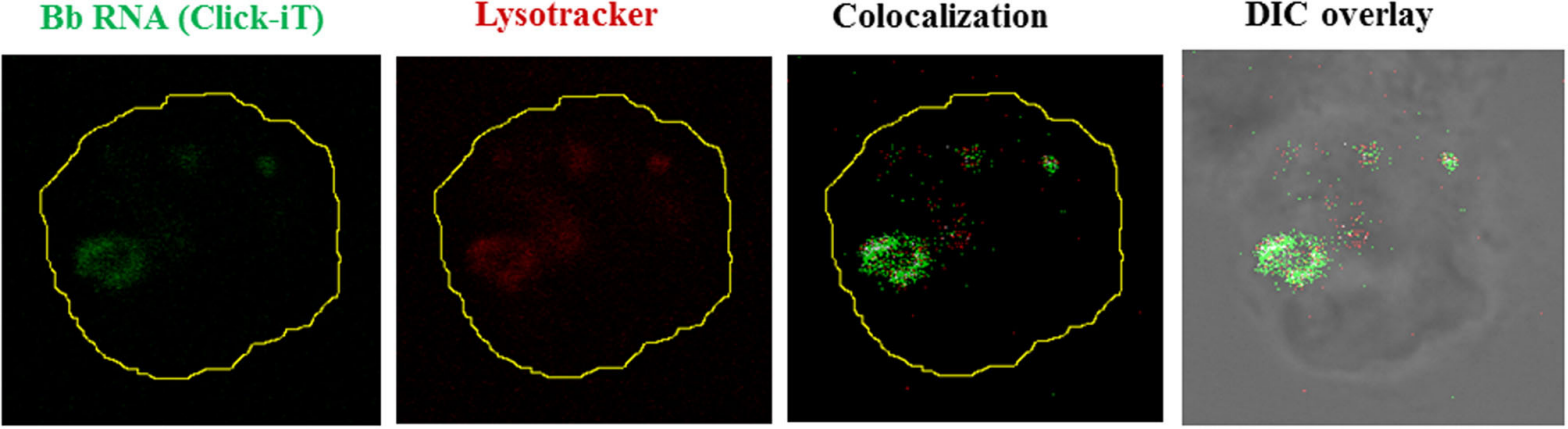
**Borrelial RNA is confined to the phagolysosome.** Live Bb whose nascent RNA has been stained with Click-iT (5 Uridine) (Green), seen internalized by a human monocyte. Lysosome stained with Lysotracker Red (Red). Colocalization shown as white pixels of the green channel colocacalizing with the red channel.

Although originally associated with the recognition viral pathogens, endosomal TLRs are also able to sense bacterial nucleic acids. The earliest evidence for such recognition was done using TLR7 and TLR8-stably transfected HEK cell lines, showing that *Escherichia coli* total RNA induced activation of TLR7 and TLR8 (Kariko et al., 2005). More recent experimental evidence confirms that TLR7 is capable of sensing bacterial RNA in both human and murine DCs, inducing the production of several NFκ-B cytokines (Eberle et al., 2009; Mancuso et al., 2009). A similar role for TLR8 activation, triggered by recognition of borrelial RNA delivered to endosomal vacuoles in human monocytes, was recently demonstrated by our group (Cervantes et al., 2013). The role of TLR8 in nucleic acid sensing was initially suggested by results from two previous studies reporting TLR8 upregulation after phagocytosis of *Mycobacterium bovis* (Davila et al., 2008), and *Helicobacter pylori* by THP-1 cells (Gantier et al., 2010). In our own studies we first showed that phagocytosis of live *Borrelia burgdorferi* by human monocytes (Figure 3) induces transcription of IFN-β (Salazar et al., 2009), and subsequently confirmed that this phenomenon was entirely dependent on TLR8 (Cervantes et al., 2011), through IRF-7; a signaling pathway traditionally associated with recognition of viral RNA (Boo and Yang, 2010).

Type I interferon responses following phagocytosis of live Bb are not restricted to IFN-β transcription by monocytes. In pDCs, production of IFN-α involves recognition of Bb ligands by TLR7 and TLR9 (Petzke et al., 2009; Love et al., 2014). In this cell type, IFN-responsive genes seem to be induced by Bb RNA through TLR7 recognition (Love et al., 2014).

Bacterial ribosomal RNA appears to be the major PAMP responsible for the production of IFN-α by human PBMCs (Eberle et al., 2009). Transfer RNA from some bacteria may also induce production of IFN-α through TLR7 activation (Jockel et al., 2012). In the case of Gram positive bacteria, such as Group B Stretptococci, bacterial ssRNA is recognized in macrophages by a TLR-MyD88-UNC93B1 complex (Deshmukh et al., 2011).

## Endosomal TLR involvement in autoimmunity and Bb nucleic acid persistence

Although the immune system has evolved mechanisms to prevent stimulation by self-nucleic acids, nucleic acid-sensing TLRs can trigger innate immune activation resulting in induction of autoimmunity (Saitoh and Miyake, 2009; Brencicova and Diebold, 2013). In fact TLR7, TLR8, and TLR9 are unable to distinguish between pathogen and self-nucleic acids on the basis of their molecular structures (Diebold et al., 2004; Heil et al., 2004; Barbalat et al., 2011). Mouse TLR7, and human TLR7 and TLR8 serve as PRR for single-stranded RNA (ssRNA), whereas the functionality of mouse TLR8 is still somewhat obscure (Cervantes et al., 2012). Presence of methylated nucleosides or pseudouridines in mammalian tRNA may also prevent TLR7 and TLR8 activation (Kariko et al., 2005). However, these nucleotide modifications are less frequent in mammalian mRNA (Maden and Hughes, 1997), which can become immunostimulatory when delivered to the endosome in form of complexes with polycations such as polyethylenimine (Koski et al., 2004; Kariko et al., 2005; Diebold et al., 2006).

TLR7 and TLR8 have already been shown to play a central role for the recognition of self RNA in the immunopathogenesis of autoimmune diseases such as systemic lupus erythematosus (SLE), psoriasis, rheumatoid arthritis, Sjögren's syndrome and others (Demaria et al., 2010; Zheng et al., 2010; Theofilopoulos et al., 2011). Human TLR8 inhibits TLR7 and TLR9 activation (Guiducci et al., 2013), and murine TLR8 also inhibits TLR7 activation (Wang et al., 2006). TLR8 deficiency leads to overexpression of TLR7 in murine DCs with increased NFκ-B activation and development of autoimmunity (Demaria et al., 2010). In humans, TLR7 and TLR9 are upregulated in patients with Sjogren's syndrome (Zheng et al., 2010). Similarly, genetic modifications that lead to a duplication of the TLR7 gene or over-expression of transgenic TLR7 are associated with exacerbated lupus-like symptoms in murine models (Pisitkun et al., 2006; Deane et al., 2007). It is worth noting that TLR7 is located on the X chromosome and that females induce higher levels of IFN-α in response to TLR7 agonists (Berghofer et al., 2006), which could represent a major factor responsible for the higher prevalence of SLE in women.

One of the more puzzling aspects of Lyme disease is the persistence, in some patients, of musculoskeletal symptoms following Bb infection, and their refractoriness to rapid improvement despite proper antibiotic treatment (Bockenstedt et al., 2012). While *erythema migrans* and Lyme carditis often present within the first few weeks of intection, Lyme arthritis more often presents several weeks after the initial infection in untreated patients and can persist even after antibiotic treatment (Kean and Irvine, 2013). Since initial studies failed to detect spirochetal DNA in human synovial fluid following antibiotic treatment in LD with persistent arthritis, also called antibiotic-resistant Lyme arthritis, it was considered an autoimmune disease (Benoist and Mathis, 2001; Steere, 2012), possibly mediated by shedding of borrelial outer surface lipoproteins (Osps) within the synovial fluid (Batsford et al., 2004). However, in recent studies, investigators were able to detect Bb DNA in joint fluid from Lyme arthritis patients who received appropriate antibiotic therapy (Picha et al., 2008; Li et al., 2011; Picha et al., 2014). Bb positive PCR results have been reported to persist for as long as 11 months in patients with antibiotic-refractory arthritis, although detection of Bb DNA did not translate into active joint disease (Li et al., 2011). DNA and antigen deposits have been shown to persist after antibiotic treatment in cartilage of mice deficient in MyD88 (Bockenstedt et al., 2012). This mouse strain exhibits more severe arthritis than WT (Bolz et al., 2004; Liu et al., 2004), and presents higher levels of IFN-β in joint tissue after infection (Petnicki-Ocwieja et al., 2013).

Naked pathogen-derived nucleic acids present in the extracellular space upon release from damaged or disintegrated microbes or the infected host cells, may be ultimately degraded by extracellular DNases and RNases before they can access the endolysosomal compartment of other immune cells (Brencicova and Diebold, 2013). If such degradation fails to occur, the presence of this remaining nucleic acid could potentially trigger an autoimmune response. This has been shown to occur with released self-DNA in SLE patients carrying mutations in DNase I (Yasutomo et al., 2001), and in DNase I-deficient mice, which develop a lupus-like disease (Napirei et al., 2000). It has been hypothesized that the sensing of naked ssRNA and DNA, which is not associated with pathogen-derived material including non-nucleic acid PAMP, doesn't allow for the discrimination between pathogen-associated vs. self-nucleic acids and, therefore, has the potential to lead to autoimmunity (Brencicova and Diebold, 2013). Mechanisms that aid in the discrimination between foreign (pathogenic) and self (cellular) nucleic acids, aim to inhibit endosomal TLR activation, or prevent cellular nucleic acid to bind to endosomal TLRs. These mechanisms include the presence of modified RNA species such as tRNA and rRNA in total cellular nucleic acids (Kariko et al., 2005), sequestration of cellular nucleic acids through binding to cellular components, and/or recruitment of nucleic acid-sensing TLR to the endolysosomal compartment or their functional activation by cleavage, a process that may be regulated by gatekeeper receptors with the ability to detect PAMP and/or DAMP absent from uninfected cells (Brencicova and Diebold, 2013). Hence, any mechanisms that allow for or promote the recognition of naked ssRNA and DNA such as in the form of immune complexes should be regarded as non-physiological events.

TLR9 is another endosomal TLR that has been linked to autoimmunity (Theofilopoulos et al., 2011), and a potential role for TLR9 in recognition of Bb DNA may exist (data not shown). Initially, it was thought that TLR9 is located in the ER in unstimulated cells and is recruited to the endolysosomal compartment only after uptake of TLR9 agonist (Latz et al., 2004). However, there is evidence of steady-state low level of trafficking of nucleic acid-sensing TLR via the Golgi to the endolysosomal compartment which may initiate TLR recruitment upon stimulation with nucleic acids (Brencicova and Diebold, 2013).

## Potential role of TLR in clinical disease severity

While TLRs are capable of sensing pathogenic materials, defective TLR signaling can hinder activation of the adaptive response. On the other hand, excessive response of TLRs and production of cytokines may increase the disease state (Kean and Irvine, 2013).

Bb produces many symptoms within the human host, including *erythema migrans*, systemic inflammation, Lyme arthritis, Lyme carditis, and neuroborreliosis (Radolf et al., 2012; Kean and Irvine, 2013). It is now known that lipoproteins can serve as potent ligands for TLRs. Bb has many Osps that are capable of triggering the innate immune system through activation of TLRs (Gondolf et al., 1994; Radolf et al., 2012). The three major Osps of Bb include OspA, OspB, and OspC. Moreover, the CD14/TLR2 complex be activated by these lipid moieties. Using a rat model Batsford et al. showed that polymerized peptide OspA produced a short lasting arthritis and that lipidated OspA and OspA elicited severe arthritis (Batsford et al., 2004). Other studies have demonstrated that macrophages play a direct role in the induction of Lyme arthritis in hamsters (DuChateau et al., 1999). These facts implicate TLRs as key players in the development of Lyme arthritis and critical receptors for recognition of vita-PAMPs.

Borrelia infection can also lead to Lyme carditis, a dangerous condition that can in rare instances can lead to sudden death (CDC, 2013). The mechanism of Lyme carditis has been shown to involve invariant NKT cells (iNKT cells) (Olson et al., 2009). This type of cell can be activated by bacterial infection through TLR4, TLR7 and TLR9-driven maturation of dendritic cells (Brigl et al., 2003). Mice deficient in iNKT cells developed significantly worse inflammation in the heart following during Bb infection (Olson et al., 2009). iNKT cells localize to the inflamed heart, enhancing macrophage phagocytosis through IFNγ leading to control of infection (Olson et al., 2009).

With recent progress in DNA analysis, the extremely polymorphic genes of TLRs can finally be understood. The genetic variability in TLRs can result in functional deficiency, which ultimately leads to immunodeficiency syndromes. The TLR1 1602S polymorphism, found predominantly in European-Caucasian populations, has been correlated with low expression of TLR1 at the surface membrane (Kean and Irvine, 2013). This mutation results in a diminished response to OspA due to the decreased capability of TLR2 to couple with TLR1, thus leading to an increased susceptibility to Lyme disease (Kean and Irvine, 2013). Individuals with mutations in the *TLR2* gene, specifically Arg753Gln, are less responsive to PAMPs derived from Bb (Schwartz and Cook, 2005).

TLR signaling alterations have been linked to more severe clinical manifestations in response to bacterial, fungal and viral infections (Frazao et al., 2013). For instance, single nucleotide polymorphisms (SNPs) found within *TLR7* have been associated with more severe Hepatits C viral infection (Lin et al., 2012). Mutations in the *TLR8* gene have been linked to increased susceptibility to bacterial infections (Davila et al., 2008). Other mutations can occur downstream of TLR-signaling. For example, deficiencies in MyD88 and IRAK4 result in impaired production of pro-inflammatory cytokines following TLR stimulation (Cervantes et al., 2013), and increased susceptibility to bacterial infections (Kenny et al., 2009; Netea et al., 2012).

It is important to note that despite the large number of TLR gene mutations found in the general population, most affected individuals will not suffer life threatening complications or even more severe infections than their unaffected counterparts (Netea et al., 2012). One explanation for the high rate of TLR polymorphism without increase in pathogen susceptibility is the redundancy of the innate and adaptive immune system. The high degree of nucleotide polymorphism seen in TLRs is consistent with the constant “arms race” driven by the rapidly evolving pathogens. Additionally, the lack of reproducibility among many experiments, in conjunction with small sample sizes of people with such phenotypes hints at the large genetic diversity among TLRs (Lin et al., 2012).

Future studies will attempt to elucidate the relationship between TLR mutations and the short and long-term outcome of human Lyme disease, particularly as to changes in these key innate immune receptors have a role in patients with prolonged, antibiotic refractory Lyme disease.

### Conflict of interest statement

The authors declare that the research was conducted in the absence of any commercial or financial relationships that could be construed as a potential conflict of interest.
